# Cobalt-Phosphate (Co-Pi)-Modified WO_3_ Photoanodes for Performance-Enhanced Photoelectrochemical Wastewater Degradation

**DOI:** 10.3390/nano13030526

**Published:** 2023-01-28

**Authors:** Jiakun Zhang, Weixu Sun, Xin Ding, Kai Xia, Tao Liu, Xiaodong Zhang

**Affiliations:** College of Chemistry and Chemical Engineering, Qingdao University, Qingdao 266071, China

**Keywords:** photoelectric catalysis, photodegradation, hydrogen production, wastewater

## Abstract

Photocatalytic technology, with features of wide applicability, mild reaction conditions and sunlight availability, satisfies the requirements of “green chemistry”. As the star photoanode material for photoelectrochemical catalysis, WO_3_ has a suitable band gap of 2.8 eV and a strong oxidation capacity, as well as displaying great potential in organic wastewater degradation. However, its performance is usually hindered by competition with water oxidation to generate peroxides, rapid charge complexation caused by surface defect sites, and so on. Herein, WO_3_ films modified with cobalt–phosphate (Co-Pi/WO_3_) film were prepared and involved in photocatalytic organic wastewater degradation. A degradation rate constant of 0.63311 h^−1^ was obtained for Co-Pi/WO_3_, which was much higher than that of WO_3_, 10.23 times that of direct photocatalysis (DP) and 23.99 times that of electrocatalysis (EC). After three cycles of degradation, the film can maintain a relatively good level of stability and a degradation efficiency of 93.79%.

## 1. Introduction

Photocatalytic organic wastewater degradation has always been a research hotspot in the field of environmental protection [1,2,3,4,5,6,7,8,9]. Photocatalytic technology, with features of wide applicability, mild reaction conditions and sunlight availability, satisfies the requirements of “green chemistry” [10]. However, the photo-generated electrons/holes in photocatalytic process are extremely easy to recombine, which affects the photon quantum efficiency and becomes the main barrier for practical application [11,12]. Sensible solutions are urgently needed in the field of photocatalytic organic wastewater degradation.

Photoelectrochemical catalysis (PEC), which can effectively promote the rapid transfer of electrons, inhibit the photogenerated electron–hole pair recombination, and facilitate the generation of strong oxidative free radicals, displays great potential in organic wastewater degradation [13,14,15]. More importantly, the catalyst is immobilized on substrate for electrodes to effectively avoid secondary pollution [16,17,18]. Since the first attempt in 1972 by Fujishima A and Honda K, photoelectrochemical catalysis has experienced rapid development involving reactors and new catalytic materials [19]. Many n-type semiconductors, such as TiO_2_, BiVO_4_, ZnO, WO_3_, etc., have been widely studied and applied in the field of photoelectric catalysis as photoanodes.

As the star photoanode material for photoelectrochemical catalysis, WO_3_ has a suitable band gap of 2.8 eV, a strong oxidation capacity [20], can capture about 12% of the solar spectrum and can absorb up to 500 nm of visible light [21,22]. The strong oxidizing substances–hydroxyl radicals can also be obtained in the water body, and the complex and refractory organic pollutants in the water body can be quickly and efficiently mineralized into CO_2_ and H_2_O for the complete mineralization of organic pollutants. However, WO_3_ also has limitations in application, such as competition with water oxidation to generate peroxides and rapid charge complexation caused by surface defect sites [23,24].

It is generally known that co-catalysts can offer certain active sites on the photoelectrode’s surface, accelerating the trapping of carriers, making it easier to separate photogenerated electron-hole pairs, and enhancing the photoelectrode’s PEC performance [25]. Cobalt-phosphate (Co-Pi) is a crucial co-catalyst among many because of its noble-free nature, capacity to speed up hydrogen precipitation reactions and improve the rate of oxygen precipitation [26,27,28,29,30,31,32,33,34,35]. The Co-Pi-modified BiVO_4_/Cu_2_O photoanode exhibits high activity and potential for methylene blue (MB) and fluoroquinolones antibiotic (CIP) degradation when used to catalyze the degradation of organic pollutants, and it is anticipated to be used consistently to catalyze the degradation of refractory organic pollutants in water [36]. In research, the Ga-doped AgInS_2_ photoanodes modified with the Co-Pi co-catalyst exhibit superior visible-light-driven PEC performance [37]. However, the assembly method of Co-Pi displays a considerable impact on the performance. Electrodeposition is a quick and efficient way to thicken the Co-Pi layer. Previous studies have shown that Co-Pi via electrodeposition is easily formed on film vacancies, fractures, or even conductive substrates. When the Co-Pi layer is too thick, the photocatalytic activity of the film is compromised [26]. By employing solely their own photogenerated holes and enabling them to be oxidized where the holes are produced, photo-assisted electrodeposition is an efficient way to prepare semiconductor photoanodes without the need for applied voltage. The photodeposition method is very effective at depositing on the most active and most hole-prone locations in the semiconductor because the photodissolution of aquatic oxygen also necessitates the consumption of photogenerated holes. With the least amount of catalyst in the photolytic water, photo-assisted deposition can, therefore, self-select the best locations for selective deposition, resulting in efficient photolytic oxygenation.

Herein, to increase the practicality of WO_3_ thin film photoanodes in photoelectrochemical cells, a substantial improvement was made in the photoelectrochemical performance and electrode stability of WO_3_ thin film photoanodes. To avoid a high-pressure environment, the WO_3_ films were first prepared using a straightforward and practical atmospheric pressure solvothermal method. A low-cost Co-Pi was further selected to modify the WO_3_ films, using photo-assisted electrodeposition as opposed to traditional electrodeposition methods, which produced a more uniform and sparser layer of Co-Pi on the semiconductor electrodes. Compared to the bare WO_3_ films, a 35.95% increase in current density was obtained at 1.23 V vs. RHE. In the degradation of methylene blue-simulated waste solution, the PEC performance of the Co-Pi/WO_3_ was 1.19 times higher than that of the pure WO_3_ films. After three cycles of degradation, the film maintained a degradation efficiency of 93.79%. Through a combination of suitable co-catalysis, our work offers an efficient method for the design and manufacture of their highly active, affordable photocatalysts.

## 2. Experimental Section

### 2.1. Materials

Analytically pure grade ethanol and hydrochloric acid reagents were purchased from Sinopharm Chemical Regent Co., Ltd. (Shanghai, China). Analytically pure grade sodium tungstate, tungstic acid, hydrogen peroxide, ammonium oxalate, and cobalt nitrate hexahydrate reagents were purchased from Shanghai Aladdin Biochemical Technology Co., Ltd. (Shanghai, China).

### 2.2. Preparation of WO_3_ Films

In the process of [38], a simple atmospheric pressure solution thermal deposition process was designed for the preparation of WO_3_ films based on the specific chemical reaction process of sodium tungstate. First, the fluorine-doped tin oxide (FTO) conductive glass was ultrasonically cleaned in acetone, anhydrous ethanol and distilled water for 30–60 min, then blown dry and set aside. An amount of 0.002 mol of Na_2_WO_4_·2H_2_O and 1.3 g/L of ammonium oxalate was weighed, dissolved in distilled water and concentrated hydrochloric acid (36% to 38%) added to the solution while stirring vigorously to ensure the pH of the solution was less than 1 [39]. According to the reaction formula $WO_{4}^{2-}+2H^{+}⇌H_{2}WO_{4}↓$, the excess hydrochloric acid causes all the tungstate ions to be precipitated in the form of tungstic acid. After stirring for a while, hydrogen peroxide (60%) was gradually added dropwise, and the tungstic acid suspension gradually became clear and transparent. This was found in the form of peroxynitric acid, which forms polyperoxynitric acid under the bridging action of oxalate- ions. Then, a 35% volume ratio of ethanol (the volume of ethanol to the volume of the solution) was added as a reducing agent to 80 mL of liquid, the conductive glass was placed conductive surface downward in a beaker, and the reaction proceeded slowly at 85 °C. After 240 min of reaction, the bright yellow film was taken out, dried at 60 °C overnight, placed in a vacuum tube sintering furnace and calcined to obtain WO_3_ films.

### 2.3. Preparation of CO-Pi/WO_3_ Films

The calcined WO_3_ film was modified with Co-Pi by the photo-assisted electro-deposition (PED) method. Specifically, the photoanode film was modified with 100 s as the unit under the three-electrode system, with 0.1 M of phosphate buffer as the electrolyte, 0.5 mM of cobalt nitrate concentration in solution and 0.1 V of voltage.

### 2.4. Characterization of Thin Films

The film structure was characterized by an X-ray diffractometer (XRD) (UltimaⅣ); scanning electron microscope (SEM) (INCA x-sight); high-resolution transmission electron microscope (HR-TEM) (FEI Tecnai G2 F20, USA); X-ray photoelectron spectroscopy (XPS) (Thermo Fisher, ESCALAB 250Xi, Waltham, MA, USA); ultraviolet-visible diffuse reflectance spectroscopy (UV-vis DRS) (Shimadzu UV3600); and energy-dispersive X-ray spectroscopy elemental analysis (EDS Mapping) (Zeiss Gemini 300, Birmingham, UK).

## 3. Results and Discussion

**Figure 1:**
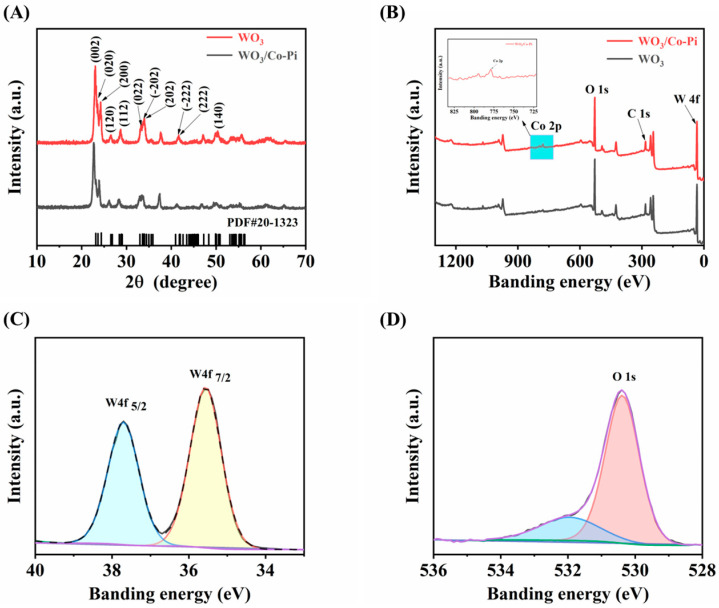
(**A**) XRD patterns of WO_3_ film and Co-Pi/WO_3_ film; (**B**) XPS patterns of WO_3_ film and Co-Pi/WO_3_ film; (**C**) W 4f XPS spectra of Co-Pi/WO_3_; (**D**) O 1s XPS spectra of Co-Pi/WO_3_.

The crystallinity of Co-Pi/WO_3_ was investigated with X-ray diffraction (XRD) patterns (Figure 1A). The eleven peaks, located at 23.05°, 23.66°, 24.34°, 26.52°, 28.76°, 33.48°, 33.7°, 34.16°, 41.26°, 42.08° and 50.04°, were ascribed to the (002), (020), (200), (120), (112), (022), (−202), (202), (−222), (222) and (140) planes of WO_3_ (PDF#20-1323), respectively. No obvious characteristic peaks for Co-Pi were revealed in the XRD patterns for low loading. X-ray photoelectron spectroscopy (XPS) tests were further executed to investigate the valence state and chemical composition of WO_3_ and WO_3_/Co-Pi. The XPS survey spectra demonstrated the presence of W, O and Co elements (Figure 1B). As shown in Figure 1C, the W 4 f_5/2_ and W 4 f_7/2_ peaks are ~37.4 eV and ~35.3 eV, and the binding energy gap between the peaks is around 2.1 eV, indicating that W is in the film at +6 valence after calcination [40,41,42]. The peak values of O1 s are given in Figure 1D, with the main body at ~530.1 eV, indicating that the chemical state was mainly present in the metal oxides, attributed to lattice oxygen, while at ~532 eV (blue part of Figure 1D) this was related to the presence of surface hydroxyl groups and ligand unsaturated oxygen species [43,44]. Initially, the surface of the prepared film was loaded with elemental Co-Pi.

**Figure 2:**
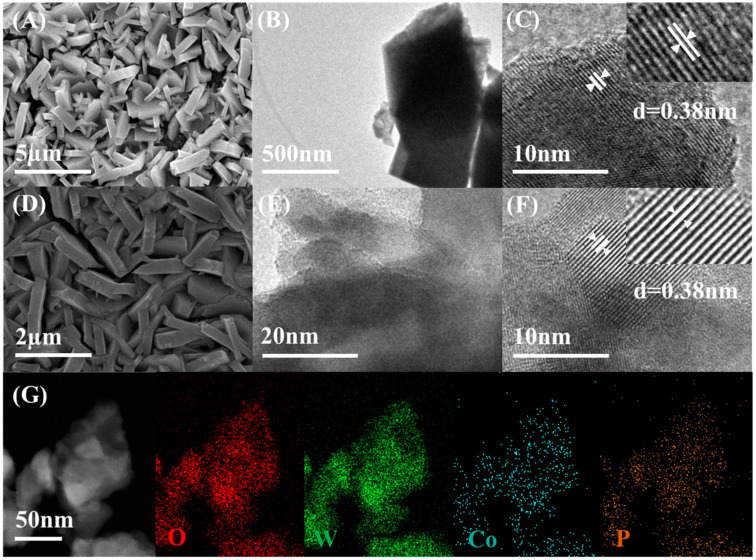
(**A**–**C**) SEM, TEM, HR-TEM images of WO_3_; (**D**–**F**) SEM, HR-TEM images of Co-Pi/WO_3_; (**G**) EDS elemental mapping images of W, O, P and Co in Co-Pi/WO_3_.

The morphologies and surface structure of WO_3_ and Co-Pi/WO_3_ were investigated by SEM and HR-TEM. As shown in Figure 2A–C, the WO_3_ films prepared and calcined by the isothermal method at atmospheric pressure were continuous and compact nanoflakes, with a measured lattice spacing of 0.38 nm for the WO_3_. Compared with the data of the standard comparison card (PDF#43-1035), the corresponding crystal plane of the material was plane (002). Surface (002) was the main exposure surface of the prepared WO_3_ film. As can be distinctly seen in Figure 2D,E, the Co-Pi-modified film had no change in morphology and was still tightly and continuously aligned with a lamellar structure. The measured lattice spacing of the WO_3_/Co-Pi films was 0.38 nm, according to the HR-TEM images (Figure 2F). No lattice spacing was detected for elemental Co-Pi for the small percentage due to the low loading, which was also similar to the published works [26]. The corresponding EDS mapping images (Figure 2G) showed the uniform distribution of W, O, P, and Co over the entire structure, which further confirms that the photoassisted electrodeposition method successfully deposited Co-Pi on the surface of the film, where it could be evenly distributed. The mass ratios of W, O, P and Co were 72.33%, 26.86%, 2.014% and 6.924%, respectively. The combination of XRD, XPS, SEM and HR-TEM results showed that the nano-sized tungsten trioxide films were prepared by the isothermal method and the Co element was successfully deposited on the surface of the films by photoassisted electrodeposition.

The photovoltaic properties were investigated using a three-electrode system in a pretreated 0.1 M NaSO_4_ electrolyte. Comparative experiments were conducted to explore the optimum process conditions for the WO_3_ films. Specifically, the photovoltaic properties of the WO_3_ films were investigated by controlling the amount of ammonium oxalate addition, reaction time, reaction temperature and ethanol dosage. As shown in Figures S1–S3, with the gradual increase in ammonium oxalate addition, the photoelectric properties showed a trend of first increasing and then decreasing. The optimum was reached at 1.3 g/L (0.54 mW·cm^−2^). In the impedance diagram (Figure S4), the resistance of the photocatalytic process showed a trend of decreasing and then increasing with the increase in ammonium oxalate addition. The reason for this change may be due to the hydrolysis of ammonium oxalate in solution to generate electron-rich groups (oxalic acid) [20], which form hydrogen bonds with the water molecules at the end of WO_3_·H_2_O and promote the orderly and continuous growth of the film. However, ammonium oxalate, as a common reducing agent, is prone to react with oxidants when heated to decomposition [45]. The addition of excess ammonium oxalate would lead to too-rapid precipitation of WO_3_·H_2_O, and some of the WO_3_·H_2_O would leak out from the bottom of the reactor in the form of precipitation, resulting in weak and discontinuous film growth, which affects the performance of the film. The reaction time and temperature control (Figures S5–S10) showed that at a reaction time of 240 min and a reaction temperature of 85 °C, the film had the lowest resistance, the highest photoresponse current and the best photovoltaic performance (0.54 mW·cm^−2^). After comparison (Figure S11), it was found that the (200) diffraction peak intensity of WO_3_ films increased and the (002) and (020) diffraction peak intensity decreased during the increase in ethanol dosage from 20 vol.% to 35 vol.%; then, when the dosage was gradually increased to 45 vol.%, the WO_3_ films’ (200) diffraction peak intensity decreased and the (002) and (020) diffraction peak intensity increased. The intensity of the (200) diffraction peak decreased and the (002) and (020) diffraction peaks increased when the dosage was gradually increased to 45 vol. The best photovoltaic performance was achieved at a specific dosage of 35 vol.% (Figures S12 and S13).

**Figure 3:**
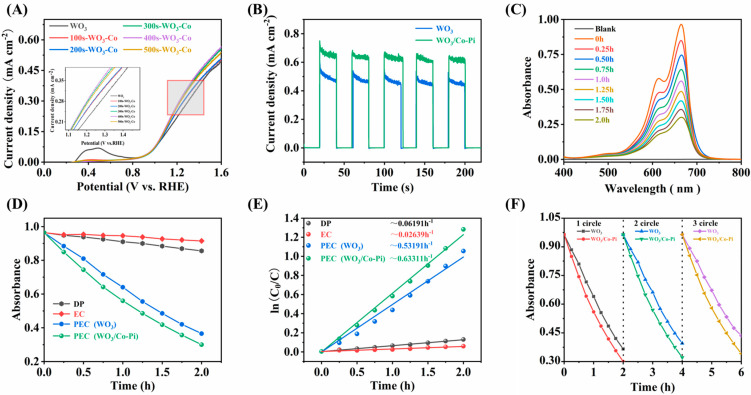
(**A**) LSV curves of WO_3_ films before and after Co-Pi modification under 100 mW·cm^−2^ light; (**B**) photoresponse current profiles of WO_3_ films before and after Co-Pi modification; (**C**) UV absorption spectra of MB degraded by Co-Pi/WO_3_ films under AM1.5 illuminations at 1 V applied voltage; (**D**) DP, EC and PEC degradation of MB under AM1.5 illuminations at 1 V applied bias voltage before and after Co-Pi modification of WO_3_ films; (**E**) corresponding kinetic curves before and after Co-Pi modification of WO_3_ films; (**F**) variation of the performance of MB degradation under PEC conditions with three cycles.

The results from the overall range of LSV plots (Figure 3A) and the photoresponse currents (Figure 3B) show that the optoelectronic properties of the Co-Pi-modified WO_3_ films were not only more stable, but also had a 35.95% increase in performance compared to the WO_3_ films alone at 1.23 V vs. improved performance. This was also confirmed by the film degradation performance tests. After exploring the optimum conditions, the photovoltaic properties of the optimized WO_3_ films were compared with those of WO_3_ films loaded with CO-Pi. The photocatalytic activity was investigated in a simulated waste solution of MB at an initial concentration of 5 mg/L using a three-electrode system. The degradation efficiency of the simulated waste solution was determined with the concentration of the simulated waste solution and the degradation rate at different times, based on the standard curve of MB staining solution (Figure S14). As shown in Figure 3C, the degradation concentration gradually decreased as the degradation time increased. After two hours of degradation, according to the degradation trend and degradation kinetic curves (Figure 3D,E), the degradation capacity of the WO_3_ film after CO-Pi modification was much greater than that of DP and EC. The highest degradation rate constant was 0.63311 h^−1^, which was 1.19 times higher than the degradation rate constant of the WO_3_ film before modification. Compared with previous works, superior performance was obtained for Co-Pi/WO_3_ (Table S1). In order to assess the stability of the films, three cycles of degradation were carried out (Figure 3F). The third time maintained a degradation efficiency of 93.79% compared to the first time and was higher than the pure WO_3_ film. This indicates that the film has good stability.

## 4. Conclusions

In conclusion, Co-Pi/WO_3_ film was prepared by light-assisted deposition, as demonstrated by XRD, XPS, SEM, TEM and HR-TEM, without altering the distinctive shape of the pure WO_3_ films involved in photocatalytic organic wastewater degradation. A degradation rate constant of 0.63311 h^−1^ was obtained for Co-Pi/WO_3_, which was much higher than that of WO_3_, 10.23 times that of DP and 23.99 times that of EC. After three cycles of degradation, the film can maintain a relatively good stability and a degradation efficiency of 93.79%. The superior performance was ascribed to the involvement of Co-Pi, which could reduce the compounding of photogenerated electron holes, increasing the efficiency of photogenerated electron transport, boosting the photocurrent and stabilizing the photoanodes. This work provides a new approach to the problem of wastewater pollution and a basis for energy conversion.

## Supplementary Materials

The following supporting information can be downloaded at: https://www.mdpi.com/article/10.3390/nano13030526/s1, Figure S1 LSV curves of WO_3_ films prepared at different ammonium oxalate contents under 100 mW·cm^-2^ light; Figure S2 Photoresponse current profiles for different ammonium oxalate additions; Figure S3 Trend of current density at 1.23V vs. RHE for films with different ammonium oxalate additions; Figure S4 Impedance mapping of different ammonium oxalate additions; Figure S5 LSV curves of WO_3_ films prepared at different reaction times under 100 mW·cm^-2^ light; Figure S6 Impedance mapping of WO_3_ films at different reaction times; Figure S7 Photoresponse current mapping of WO_3_ films at different reaction times; Figure S8 Trend of current density at 1.23V vs. RHE for WO_3_ films at different reaction times; Figure S9 LSV curves of WO_3_ films prepared at different reaction temperatures under 100 mW·cm^-2^ light; Figure S10 Impedance diagram of WO_3_ films at different temperatures; Figure S11 XRD patterns of WO_3_ films prepared at different ethanol dosages; Figure S12 LSV curves of WO_3_ prepared at different ethanol dosages under 100 mW·cm^-2^ light; Figure S13 Impedance diagram of WO_3_ films with different ethanol dosages; Figure S14 UV-Vis absorptions spectra (A) and calibration curve (B) used for calculation of methylene blue staining solution.
