# Supplementary material for: Cobalt-Phosphate (Co-Pi)-Modified WO3 Photoanodes for Performance-Enhanced Photoelectrochemical Wastewater Degradation
Source: Nanomaterials (Basel). 2023 Jan 28;13(3):526. doi: 10.3390/nano13030526 (PMC9921962; doi:10.3390/nano13030526)
Supplement: Supplementary file 1 [file nanomaterials-13-00526-s001.zip › nanomaterials-2152615-supplementary.pdf]

Support Information

Article

Cobalt–Phosphate (Co-Pi)-Modified WO_3 Photoanodes for Performance-Enhanced Photoelectrochemical Wastewater Degradation

Jiakun Zhang †, Weixu Sun †, Xin Ding *, Kai Xia, Tao Liu * and Xiaodong Zhang *

College of Chemistry and Chemical Engineering, Qingdao University, Qingdao 266071, Shandong, China; zhangjiakun@qdu.edu.cn (J.Z.); sunweixu@qdu.edu.cn (W.S.); xiakai@qdu.edu.cn (K.X.);

* Correspondence: dingxin@qdu.edu.cn (X.D.); liutao@qdu.edu.cn (T.L.); zhangxiaodong@qdu.edu.cn (X.Z.)

† These authors contributed equally to this work.

Light response current and its stability testing

The silver/silver chloride electrode R0305 (Aida Hensheng, Tianjin) was used as the reference electrode, the prepared photoanode as the working electrode and the platinum mesh electrode as the counter electrode. The test was carried out with a 0.1 mol/L concentration of $NaSO_4$ solution as the electrolyte, pH=6.5, a xenon lamp with a UV filter (>400 nm) as the light source, the light intensity was calibrated to 100 $mW\cdot cm^{-2}$ by an irradiation meter (FZ-A type) and the test was carried out under the control of an electrochemical workstation (CHI 760E) with a test voltage of 1.23 V vs. intervals of 0.01. All potentials were measured using the Ag/AgCl reference electrode and converted to the RHE reference scale using the equation: $E_{RHE} = E_{SCE} + 0.0591 \times pH + E^{0}_{SCE}$.

Linear scanning voltammetry

The photocatalytic activity of different working electrodes was measured relative to darkness in the range 0-1.6 V vs. RHE using a $NaSO_4$ solution at a concentration of 0.1 mol/L, pH=6.5 as the electrolyte. The scanning rate was 5 mV/s and the sampling interval was 0.001 V.

AC impedance testing

Tests were carried out with a 0.1 mol/L concentration of $NaSO_4$ solution as the electrolyte solution, under light, 1.23 V vs. RHE voltage, 1,000,000 Hz at high frequencies and 0.1 Hz at low frequencies, with an amplitude of 5 mV.

Photocatalytic activity testing

The degradation rate of MB was measured based on the absorbance of the initial organic pollutant MB at the position of the maximum absorption peak (664 nm) in the UV-Vis absorption spectrum. The initial concentration of MB was 5 mg/L , and the test was taken at 15-minute intervals with the working electrode at a light intensity of 100 $mW\cdot cm^{-2}$. During the test, the solution was left in a stirred state and the working electrode was placed in an electrolytic cell in a dark environment for 30 minutes to ensure equilibrium of MB adsorption and desorption on the working electrode before testing the MB degradation efficiency. The change in absorbance of the simulated waste solution was measured using a UV-Vis spectrophotometer.

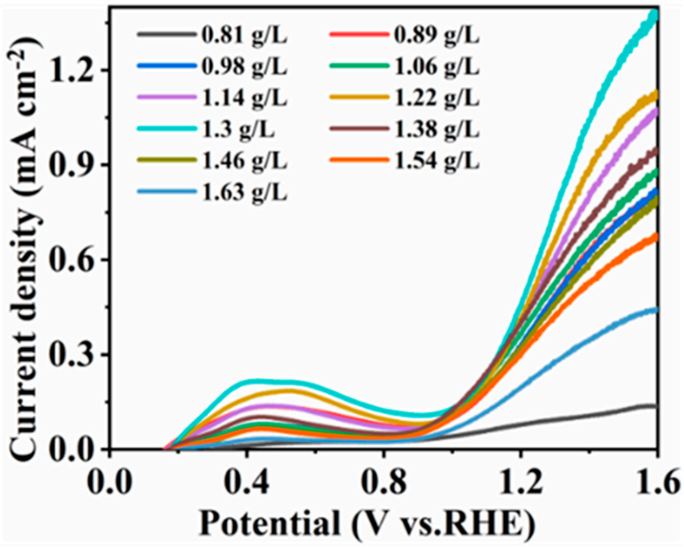

Figure S1. LSV curves of WO_3 films prepared at different ammonium oxalate contents under 100 mW·cm^{-2} light.

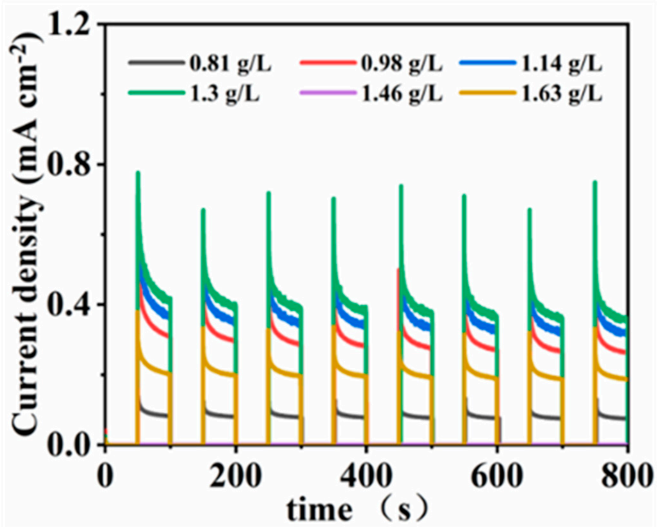

Figure S2. Photoresponse current profiles for different ammonium oxalate additions.

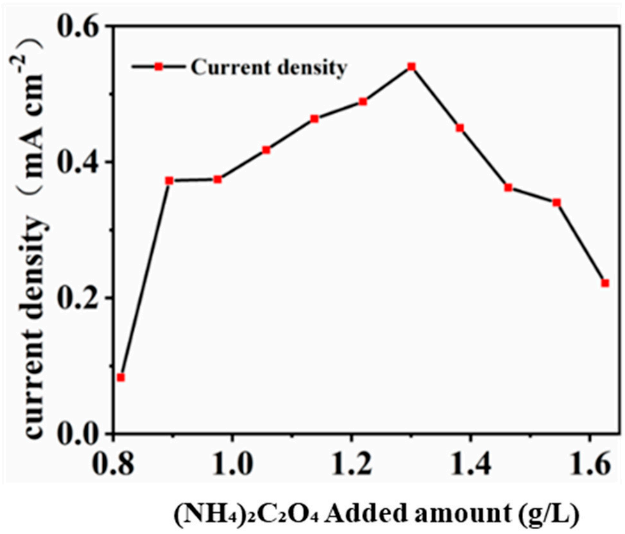

Figure S3. Trend of current density at 1.23V vs. RHE for films with different ammonium oxalate additions.

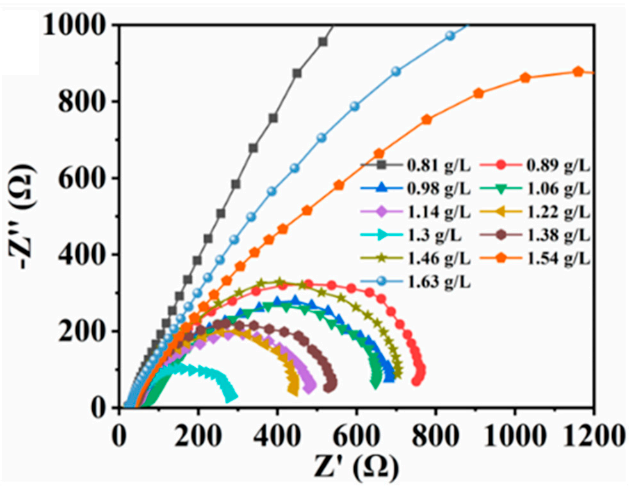

Figure S4. Impedance mapping of different ammonium oxalate additions.

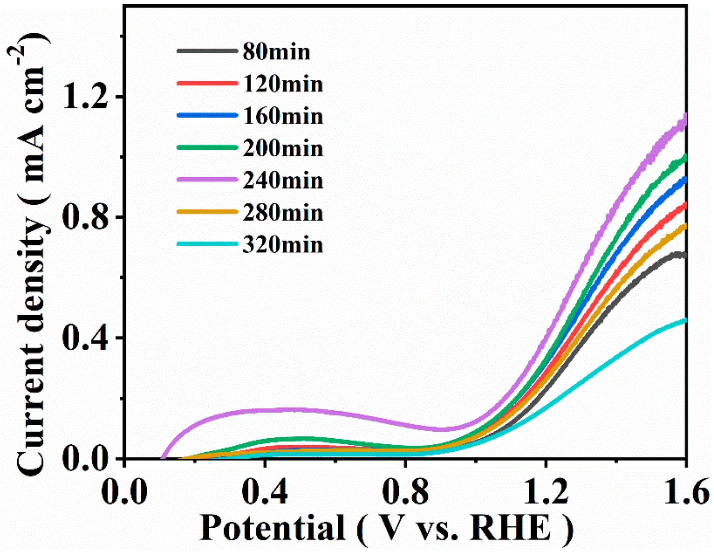

Figure S5. LSV curves of WO_3 films prepared at different reaction times under 100 mW·cm^{-2} light.

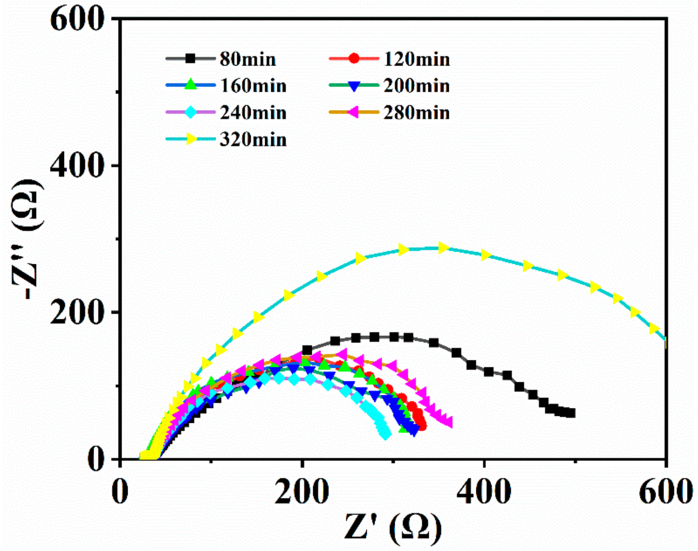

Figure S6. Impedance mapping of WO_3 films at different reaction times.

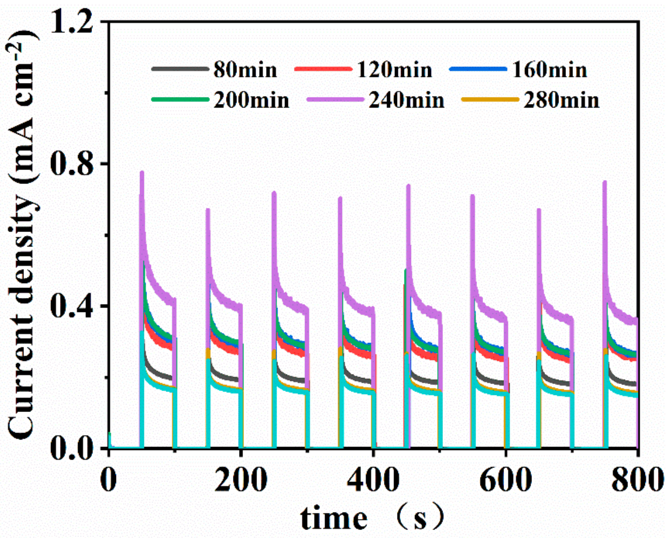

Figure S7. Photoresponse current mapping of WO_3 films at different reaction times.

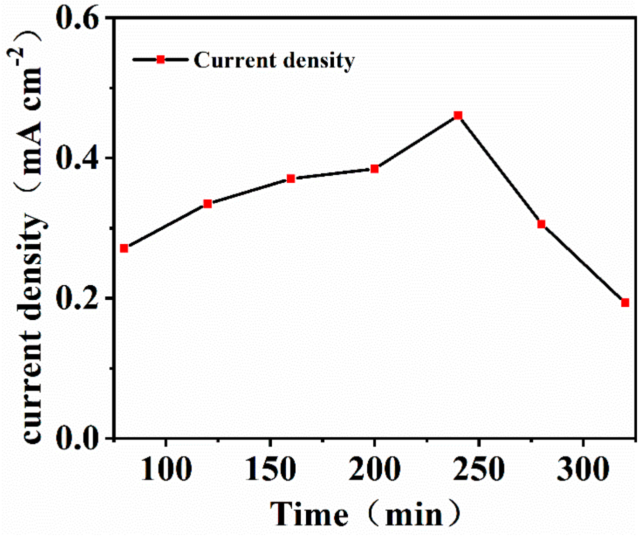

Figure S8. Trend of current density at 1.23V vs. RHE for WO_3 films at different reaction times.

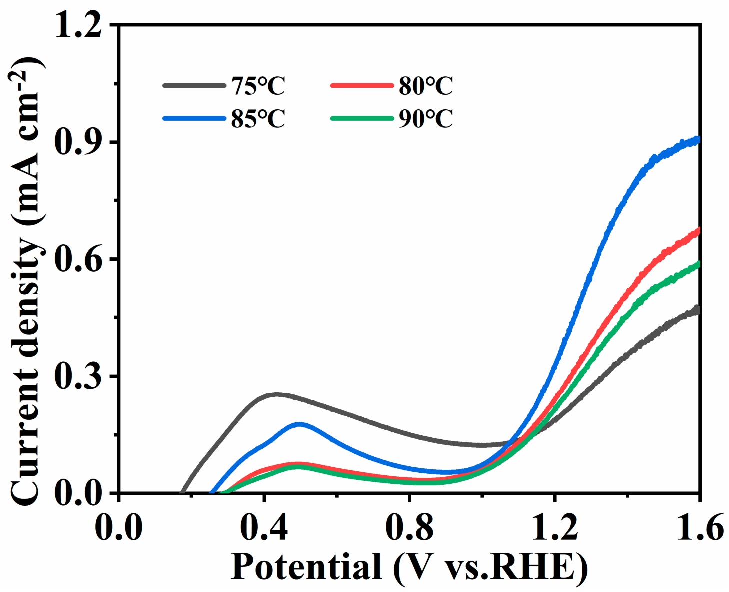

Figure S9. LSV curves of WO_3 films prepared at different reaction temperatures under 100 mW·cm^{-2} light.

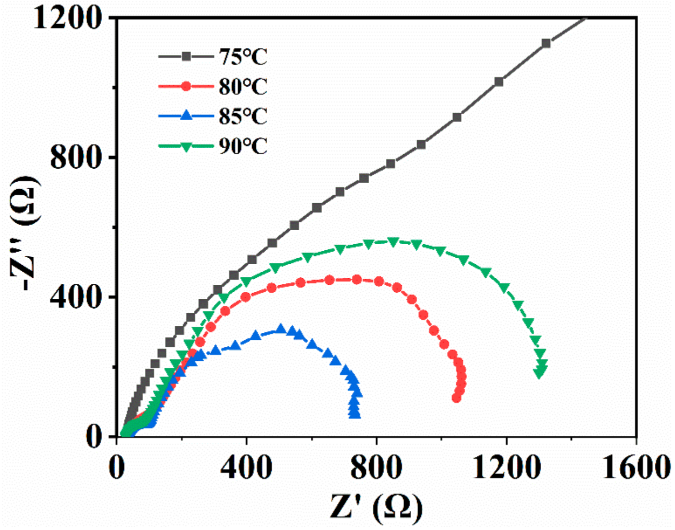

Figure S10. Impedance diagram of WO_3 films at different temperatures.

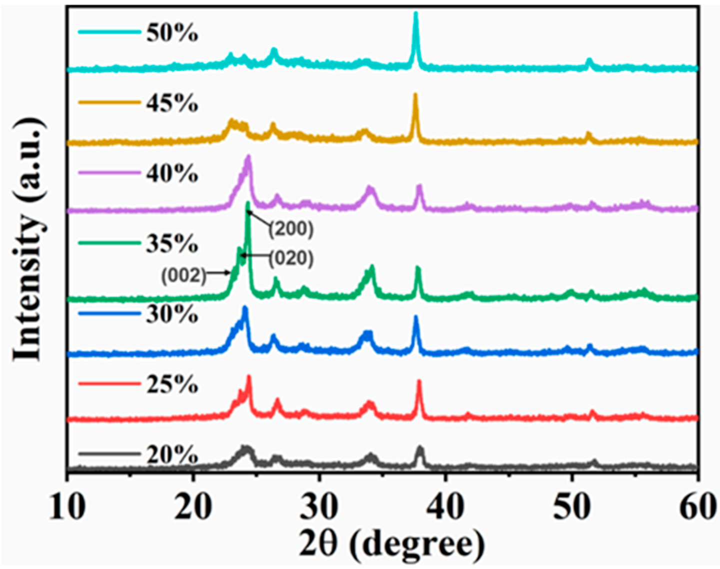

Figure S11. XRD patterns of WO_3 films prepared at different ethanol dosages.

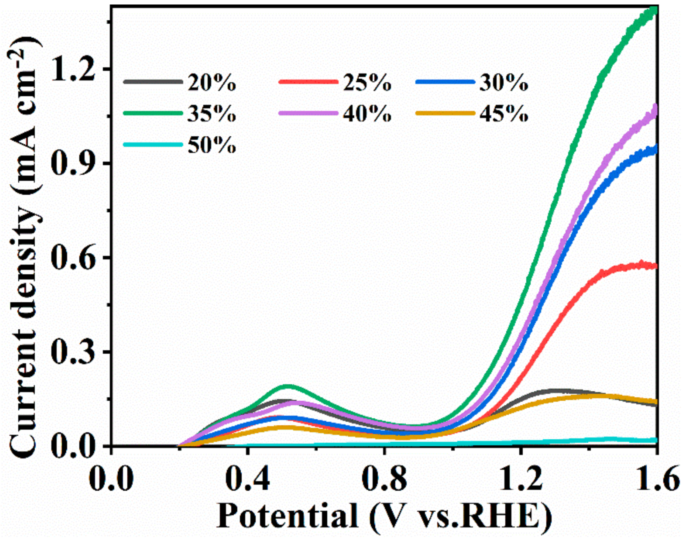

Figure S12. LSV curves of WO_3 prepared at different ethanol dosages under 100 mW·cm^{-2} light.

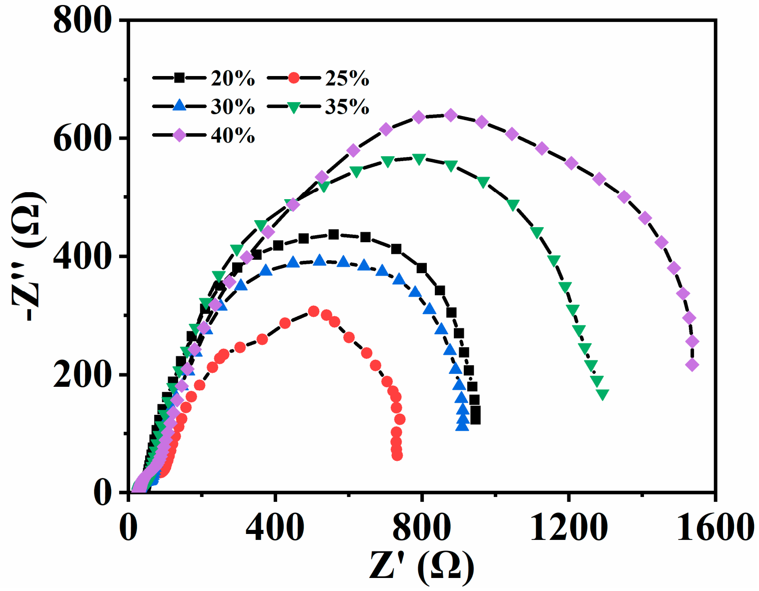

Figure S13. Impedance diagram of WO_3 films with different ethanol dosages.

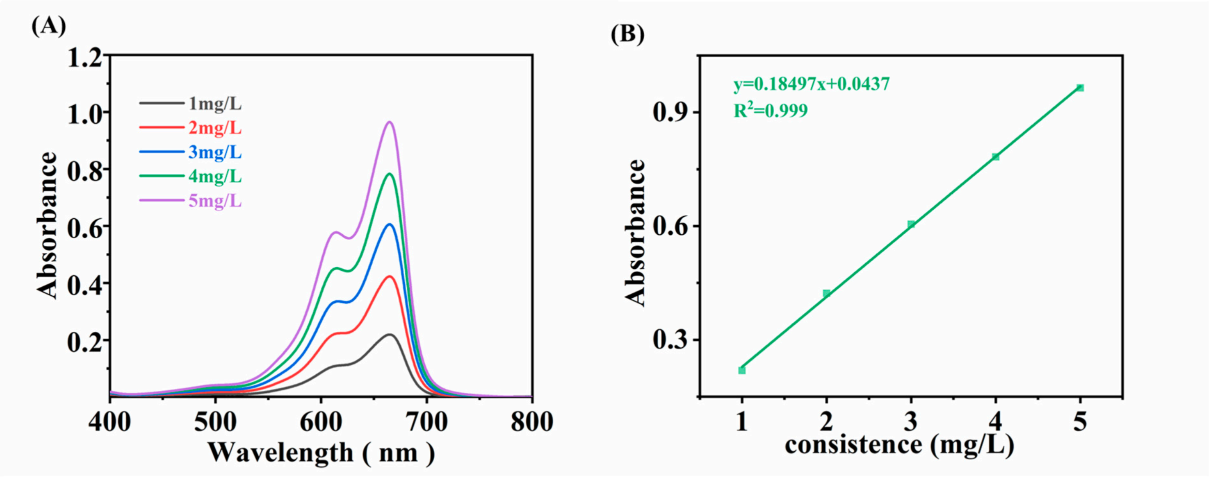

Figure S14. Standard curve of methylene blue staining solution.
